# The web of clinical data, bioengineering, augmented reality and robotic in vascular surgery

**DOI:** 10.3389/fsurg.2022.971776

**Published:** 2022-10-14

**Authors:** Carlo Setacci, Alberto Maria Settembrini, Domenico Benevento

**Affiliations:** ^1^ Vascular Surgery Unit, University of Siena, Siena, Italy; ^2^ Unit of Vascular Surgery, Foundation IRCCS Ca’ Granda Ospedale Maggiore Policlinico, Milan, Italy

**Keywords:** bioengineering, vascular, robot, artificial intelligence, hybrid procedure, augmented reality

The new protocol for information distribution amongst scientists within the CERN, once slightly refined and publicly announced as the Worldwide Web in the August of 1991, has led to impressive achievements beyond the starting plan.

Let’s imagine a similar story: we replace the scientists who study particle physics at CERN with scientists and professionals in medicine and healthcare to create the Web of Clinical Data (WCD). Like at CERN, only persons with proper authentication credentials could access the WCD, which would become a huge repository of personal health records (PHR) and clinical data from medical centers worldwide. Scientists and professionals in medicine could gain access to the repository by applying to an international scientific committee that supervises the WCD.

Doctors could access anonymous PHR located in any corner of the world employing software tools similar to those we daily experience on the Web. The WCD would become a paradigm shift (1) in the way students and experts in medicine access clinical data, and it could open the doors to the grand challenge of building decision support systems for medical decisions. These software agents could exploit the content of the health records and their similarities, so successful treatments buried in remote corners of the planet could be retrieved based on automatic induction from similar patterns of clinical data. In addition to the direct access to word, WCD offered to scientists and medical professionals, the progressive growth of the WCD would also catalyze emerging technologies in the broad field of artificial intelligence, with an enormous impact on medicine.

While this colorful story, which crosses fiction, makes us dream of fantastic progress in medicine and a massive diffusion of successful health care protocols, especially in third world countries, one might be overwhelmed by anxiety and fears of violating our privacy. Although in good faith, you may be tempted to dampen down these terrible Orwellian distortions and switch them off vigorously before they can unleash their contaminant force. However, the history of science teaches us that everything imaginable, sooner or later, will be the object of attention and study and that no proclamation can extinguish such a curiosity. History teaches us not to evoke stale forms of luddism, that freedom has a high price, and that man is called to govern the application of nuclear physics to produce energy rather than to construct nuclear weapons. Interestingly, this story of the WCD is only partially new, and those privacy issues have already been carefully considered. In the United Kingdom, a few years ago, the NHS National Institute for Health Research (NIHR) and the Medicines and Healthcare products Regulatory Agency (MHRA) jointly funded the Clinical Practice Research Datalink (CPRD). It is a new English NHS observational data and interventional research service designed to maximize the link of anonymous clinical data to originating research activities. It aims to generate outputs that are beneficial to improving and safeguarding public health.

Since the earliest work on Artificial Intelligence (AI) in medicine in the 1970s, advances in Technology and computational methods have led to a growing interest in potential applications in both Medical Research and Clinical practice. According to Vuong et al., the use of AI in medicine can be categorized into two main branches: the physical branch, which includes the development of assistive robots for care, surgery, or drug delivery, and the virtual branch, including the development of the informatics approach and expert systems (2, 3).

About augmented reality, recently, this technology has been successfully helping surgeons during image-guided integration of surgical navigation with virtual planning simultaneously with the real patient anatomy.

In open surgery augmented reality is important in surgical planning and patient specific study to navigate before complex operations and in simulation systems for training.

On the other side, in endovascular surgery evaluation of the potential benefits of wearable displays for performing fluoroscopically guided interventional procedures is currently being explored for developing innovative intraoperative platforms to assist the surgeon during the navigation (4, 5).

To date, despite the reported series by a few pioneers, laparoscopic aortic surgery has not been widely embraced by the majority of vascular surgeons.

This lack of interest can probably be explained by the technical difficulties experienced by surgeons with endoscopic techniques, especially during the performance ho aortic anastomosis (6–10).

This technical difficulty in suturing vascular anastomoses with laparoscopic instruments, as the main limitation, has stimulated the development of hybrid procedures whereby laparoscopic dissection is followed by hand-sewn anastomosis through a mini-laparotomy incision or and to hand-assisted laparoscopic surgery (HALS). However, laparoscopy's maximal benefit is achieved by avoiding mini-incisions altogether.

The application of robotic techniques in vascular surgery can be considered a great innovation and an actual revolution in the surgical field. This technique provides certain advantages for some surgical interventions, especially during specific tasks of the procedure, in light of its endo-effectors linked to mechanical arms that enable incredibly precise movements, considered practically impossible by direct human manipulation (11).

The slow adoption of robotic vascular surgery can also be attributed to the perceived risk associated with the telemanipulation of vessels. However, several published series of partial and total robotic aortoiliac operations have demonstrated low mortality and acceptable conversion rate (12–14).

Aortoiliac surgery, more aneurysmal disease than the occlusive, is undoubtedly a good indication to perform mini-invasively aortoiliac reconstructions with the robot, offering better clinical outcomes for patients than open surgery. There is still a place for conventional repair besides endovascular techniques, and life expectancy will benefit most from robotic surgical repair of abdominal aortic aneurysm repair.

A close collaboration between Doctors and Data scientists plays a crucial role in developing fitting tools and guiding engineers to answer the right medical question with the correct data (15).
